# HPLC Quantification of Phenolic Acids from *Vetiveria zizanioides* (L.) Nash and Its Antioxidant and Antimicrobial Activity

**DOI:** 10.1155/2013/270472

**Published:** 2013-03-11

**Authors:** Jha Prajna, Jindal Richa, Chakraborty Dipjyoti

**Affiliations:** Department of Bioscience and Biotechnology, Banasthali Vidyapith, Rajasthan 304022, India

## Abstract

Extraction procedure was standardized and for the soluble, glycoside, and wall-bound fractions of phenolic acids from *Vetiveria zizanioides*. The water soluble alkaline extract which represents the cell wall-bound fraction contained the highest amount of phenolic acids (2.62 ± 1.2 *μ*M/g fwt GA equivalents). Increased phenolic content in the cell wall indicates more lignin deposition which has an important role in plant defense and stress mitigation. Antioxidant property expressed as percentage TEAC value obtained by ABTS assay was correlated with the amount of phenolic acids and showed a Pearson's coefficient 0.988 (significant at 0.01 level). The compounds *p*-coumaric acid, *p*-dihydroxybenzoic acid, and ferulic acid were detected in the acidic extracts by HPLC analysis. The plant extracts exhibited considerable antimicrobial activity against tested bacterial and fungal strains.

## 1. Introduction

Natural products play a dominant role in pharmaceutical industry, and systematic investigation of natural resources for the discovery of new drug molecules is the primary objective for bioprospection programs [1]. There is about 127 natural products or natural-product-derived compounds currently undergoing clinical trials [2]. The common phenylpropanoid pathway is an essential pathway of secondary metabolism leading to a diverse range of compounds that are important both to the physiology of plants and to the many applications of plant natural products from lignin to pharmaceuticals and nutraceuticals [3, 4]. Microbial resistance to antibiotics is a global concern covering all known classes of natural and synthetic compounds fueling the need for novel antimicrobials of which plants are a potent source [5–7].

*Vetiveria zizanioides* (L.) Nash (Poaceae), a perennial grass grows abundantly in the Gangetic plains of India and holds an esteemed place in the Indian mythology and traditional medicinal practices [8]. It is used as a relaxant for the nervous system, lowers heart rate, normalizes breathing, has anti-inflammatory properties, controls diabetes, and cures skin diseases [9]. The root decoction of the plant is used as an analgesic and, in inflammation, as an anthelmintic [10], antipyretic [11], and antioxidant [12], and as an antituberculosis agent [13]. The leaf juice is used as anthelmintic [14] and applied as paste for relief from rheumatism, lumbago, and sprain [15]. Cultivation of Vetiver grass has ecological advantages as it decreases soil erosion and is recommended in conservation studies [16]. Leaves of the plant are also used as raw material for handicraft making, mushroom cultivation, roof thatching, and several industrial products [17]. Although several bioactive compounds such as essential oils, stilbene derivatives, lipids, and phytosterols are reported from the plant [18], there is no information on phenolic compounds. Considering that the leaves provide more biomass as compared to the roots and can be regularly harvested, the present study aims to evaluate the antimicrobial activities of the leaf extracts with emphasis on phenolic compounds.

## 2. Materials and Methods

### 2.1. Plant Material

*V*. *zizanioides* plants used for the present study were collected from Darbhanga district, North Bihar, India and authenticated by Dr. V Jha, Department of Botany, CM Science College, Darbhanga, India.

### 2.2. Test Organisms

The bacterial strains *Escherichia coli* and *Bacillus subtilis* were obtained from IMTECH, Chandigarh, India. *Micrococcus luteus* was obtained from the Microbiology Laboratory, Banasthali Vidyapith, India. The fungal strains (*Aspergillus niger*, *Fusarium oxysporum*, and *Macrophomina phaseolina*) were obtained from the Plant Pathology Laboratory, Banasthali Vidyapith, India.

### 2.3. Chemicals

The phenolic acid standards, *p*-coumaric acid hydroxybenzoic acid, and ferulic acid were obtained from Fluka, Chemie GmBH. ABTS was obtained from Sigma Aldrich. HPLC-grade methanol and water were procured from Merck, India.

### 2.4. Extraction of Phenolic Acids

Extraction of phenolics was done for alcoholic (80% methanol (M)), acidic (0.1 N HCl, water soluble (HW), methanol soluble (HM)), and alkaline (1 N NaOH, water soluble (NW), methanol soluble (NM)) extracts following the previously published protocols [19–23]. In alcoholic extraction process, leaves (1 g) were crushed in 5 mL of 80% aqueous methanol, filtered, and centrifuged at 5000 rpm for 10 min. The supernatant was collected. For aqueous acidic extract, leaves (1 g) were boiled in 0.1 N HCL for 25–30 min and filtered. The filtrate was then partitioned with ethyl acetate and dissolved in water (HW) and the portion insoluble in water dissolved in 80% methanol (HM). In the alkaline extraction process, leaves (1 g) were boiled in 0.1 N HCL for 25–30 min and centrifuged at 5000 rpm for 10 min. The residue was kept in 1 N NaOH (2 mL) overnight. It was again centrifuged and the filtrate brought to pH 2, partitioned ethyl acetate and dissolved in water (NW) and the portion insoluble in water dissolved in 80% methanol (NM).

### 2.5. Determination of Total Phenolic Content

Freshly harvested leaves were used for analysis. Total phenolics were determined with Folin-Ciocalteu reagent, which takes into account all hydroxylated aromatic compounds and expressed as gallic acid equivalents. The phenolic content was estimated by taking absorbance at 660 nm using a Systronics 2269 Spectrophotometer (Systronics, India) and expressed as gallic acid (GA) equivalents.

### 2.6. HPLC Analysis of Phenolic Acids

For quantitative estimation of phenolic acids, HPLC was performed using a RP C18 Whatman (UK) 5 ODS column (25 cm × 4.6 mm) on a Shimadzu HPLC system (System Control SCL: 10A, Uv-vis detector: SPD 10A, Pump: LC-10AT, Shimadzu, Japan) using a modified method of Sachan et al. [24]. The polar mobile phase comprised with methanol: aqueous 1 mM of trifluoroacetic acid 30 : 70 at a flow rate of 1 mL min^−1^ for 20 min and the detector monitored at 254 nm. To minimize variation in quantification, samples were taken in triplicate.

### 2.7. Total Antioxidant Activity 

*ABTS Assay*. The ability of the test sample to scavenge ABTS+ radical cation was compared to trolox standard [25]. The percentage inhibition of absorbance on test sample on ABTS was calculated and plotted as a function of the concentration of standard and sample to determine the trolox equivalent antioxidant concentration (TEAC). All experiments were repeated three times.

### 2.8. Antibacterial Assay

Antibacterial activity of phenolic extracts was tested using broth dilution method following [26]. Briefly, an aliquot of individual bacterial stock was grown overnight at 37°C in 2 mL liquid LB medium for inoculation. Inoculum was added to culture medium supplemented with various concentrations (100, 200, and 300 *μ*L) of extract. Bacterial culture without extract was used as control and growth was measured at 540 nm after 24 h. Kanamycin (10 *μ*g/mL) was used as a positive standard in the assay.

### 2.9. Antifungal Assay

Phenolic acid extracts were added to Potato Dextrose Broth medium (50 mL) in conical flasks. The mycelia of fungal strains were transferred to the flasks and incubated at 37°C. When the fungal mycelium reached the edge of the control flask, the dry weight was determined by filtering and drying in an oven at 50°C. Bavistin was used as a positive standard in the assay. Antifungal index was calculated by the formula:

(1) $$\begin{array}{c} \text{Antifungal}\;\text{index}\;\left(\text{AI}\%\right)=1-\left(\frac{{\text{W}}_{\text{a}}}{{\text{W}}_{\text{b}}}\right)*100, \end{array}$$

where W_a_ is the weight of the fungal mycelia in the experimental set and W_b_ is the weight of the mycelium in control set.

### 2.10. Statistical Analysis

A randomized block design was used to set up all the experiments. Data were examined by analysis of variance (ANOVA) to detect the differences (*P* = 0.05) between the means and compared using Tukey's HSD test at the same (5%) probability level using SPSS software (ver. 17.0.0; SPSS, Chicago, IL, USA). LC_50_ values were computed by Probit analysis in R-Software (R version 2.9.0, The R Foundation for Statistical Computing, 2009). Correlation between phenolic content and antioxidant value was done by Pearson's coefficient.

## 3. Results and Discussion

The aim of the present work is to standardize extraction procedures for phenolic acids from *V*. *zizanioides* and investigate its antioxidant and antimicrobial activity.

The concentrations of total phenolics and the antioxidant property of the different fractions of plant extracts are represented in Table 1. It was observed that the phenolic content varied according to the extraction procedure for optimum release of the metabolites from the leaf matrix. Alcohols (methanol, ethanol) are frequently used to extract phenolic compounds from plant material [19, 20]. In *V*. *zizanioides*, higher amounts of phenolic acids were extracted by the acidic and alkaline extraction procedure as compared to methanolic extraction indicating the presence of glycoconjugates. Saponification of the residue left after acidic extraction with NaOH (NM and NW) that releases cell wall-bound compounds resulted in highest yield of total phenolics in *V*. *zizanioides*. Several simple phenylpropanoids (with basic C6–C3 carbon skeleton) are rarely found in the free form in plant cells and are generally conjugated to sugars, cell wall carbohydrates, or organic acids [21, 22]. The presence of such conjugates leads to extraction of higher amount of phenolic acids on acid/alkali treatment. Plant matrices behave differentially to extraction solvents for phenolic acids, and a compound may not form the same conjugate in another species necessitating standardization of optimum extraction procedure [23].

**Figure 1:**
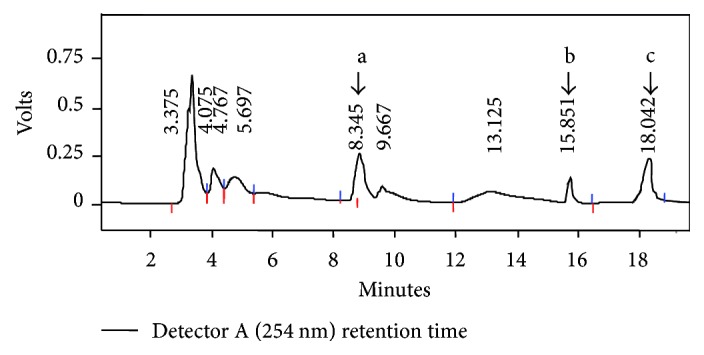
HPLC chromatogram of the phenolic acids (acidic fraction) from *V*. *zizanioides* leaves. The values represent retention time (*R*_*t*_) in min. a: *p* hydroxybenzoic acid, b: *p* coumaric acid, and c: ferulic acid.

HPLC analysis of the acidic extract showed eight peaks (Figure 1) out of which three were identified as *p*-hydroxybenzoic acid (*R*_*t*_ 8.34 min, 5.58 *μ*g/g fresh weight), *p*-coumaric acid (*R*_*t*_ 15.83, 13.2 *μ*g/g fresh weight), and ferulic acid (*R*_*t*_ 18.608, 14.17 *μ*g/g fresh weight). *R*_*t*_'s of the peaks that were not identified are as follows: 4.13, 4.93, 6.57, 9.13, and 16.95. Hydroxycinnamates such as *p*-coumaric acid are major components of plant cell walls, particularly of monocots and are esterified with lignin—a key cell wall component [27, 28]. Hydroxybenzoides have widespread application in food and pharmaceutical industry [29] and are also important intermediates in synthesis of fragrance and flavor compounds in plants [30]. Recently, it was shown that certain rot fungi have the capacity to convert *p*-coumaric to *p*-hydroxybenzoic acid [31] which has considerable ecophysiological and commercial implications. Ferulic acid has an important role in plant cell wall formation [32] and is an effective antioxidant [33] and anticancer compound [34]. Commercially ferulic acid is synthesized from petroleum. There are few commercially viable biological sources of ferulic acid such as rice barn [35], and *V*. *zizanioides* might offer a cost effective alternative.

The alkaline water soluble extract, which pertains to the cell wall component of *V*. *zizanioides*, had the highest antioxidant value expressed as percentage TEAC (Table 1). Pearson's correlation coefficient value was found to be 0.988 (significant at *P* = 0.01) indicating that phenolic acids present in the different fractions highly correlate with the TEAC values. Thus, antioxidant property of the extracts can be attributed to the presence of phenolic acids. Vetiver oil is reported to possess a strong free radical scavenging activity when compared to standard antioxidants such as butylated hydroxytoluene (BHT) and *α*-tocopherol [36]. High antioxidant potential of the hexane extracts obtained from roots of Vetiver grass is also reported [37].

**Figure 2:**
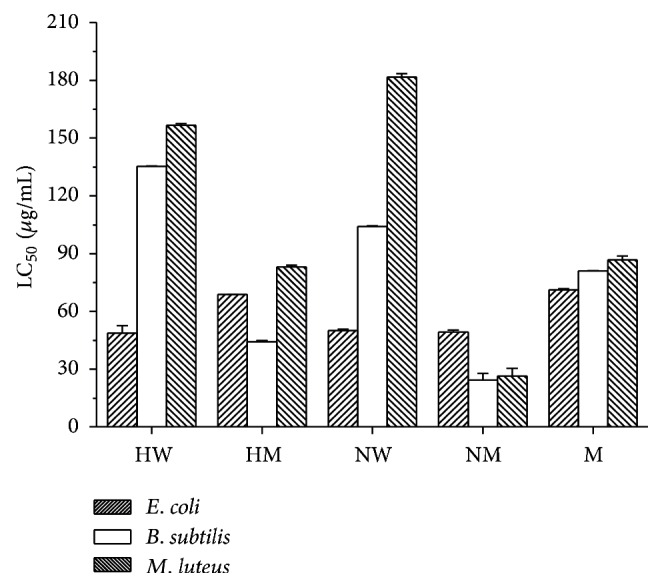
Antibacterial activity (LC_50_ values) of the phenolic extracts of *V*. *zizanioides* against the bacterial strains *E*. *coli*, *B*. *subtilis*, and *M*. *luteus*. HW: water soluble acidic fraction, HM: methanol soluble acidic fraction, NW: water soluble alkaline fraction, NM: methanol soluble alkaline fraction, and M: 80% methanolic extract.

The antibacterial activity of the plant extracts is represented in Figure 2. Initially the crude phenolic extracts were tested for their effectiveness against the bacterial strains in agar plate assay (results not shown). The quantification of antibacterial activity was done by broth assay method. In general, the extracts were more potent against the gram negative *E*. *coli* in comparison to *B*. *subtilis* and *M*. *luteus*, except for the alkaline methanol soluble fraction which was more potent against *M*. *luteus* and the methanol soluble acid extract which was more active against *B*. *subtilis*. The water soluble and methanol soluble alkaline extract showed the lowest LC_50_ values hence highest activity against *E*. *coli*. Alkaline extract dissolved in methanol showed highest activity against *B*. *subtilis* and *M*. *luteus*. The LC_50_ values of Kanamycin against *E*. *coli*, *B*. *subtilis*, and *M*. *luteus* were 146.36, 121.18, and 45.42 *μ*g/mL, respectively.

The water soluble alkaline fraction contained the highest amount of phenolic acids and it was the most effective against the *B*. *subtilis* and *M*. *luteus*; however, the methanolic fraction although contains lower amount of phenolic acid is more effective against *E*. *coli* in comparison to the other extracts. The lowest concentration of phenolic acid was extracted in the methanol soluble acidic fraction, but the minimum activity was shown by the methanol soluble alkaline fraction, indicating the importance of the type of phenolic acids to be more important for activity rather than the amount. The methanol soluble alkaline fraction was also less active against gram positive bacteria as compared to gram negative bacteria, a feature which is not observed in the other extracts except for methanol soluble acidic fraction. The wide variation in the activity of different phenolic extracts of the same plant species indicates the variation of the phenolic constituents extracted from the plant matrix by acidic, alkaline, and alcoholic extraction procedures.

The antifungal activity was calculated as antifungal index with shows the percentage of growth inhibition of the fungi (Table 2). In contrary to the antimicrobial activity, the methanol soluble alkaline fraction consistently showed better antifungal activity against *A*. *niger* and *M*. *phaseolina*. The water soluble acidic fraction and the methanolic fraction were more effective against *F*. *oxysporum* although the content of total phenolics of methanol soluble alkaline fraction is lower than the water soluble acidic fraction.

Similarly, although the total phenolic content of the methanol soluble acidic fraction is lowest among all the extracts, it shows potent antifungal activity against all the fungal strains tested in contrast to the water soluble acidic fraction which is less effective against *A*. *niger* and *M*. *phaseolina*. It is, thus, evident that a simple correlation between the total phenolic content and its antibacterial/antifungal activity cannot be drawn, rather the individual constituents of phenolic acids and their concentration variation have to be taken into consideration.

The antimicrobial activity observed *in vitro* against the three bacterial and three fungal strains could be attributed to the presence of polyphenolic compounds in the *Vetiveria* extracts. It is postulated that the antibacterial properties of phenolic acid are due to nucleoid damage with an increase in spatial division and condensation of genetic material [26]. Phenolic acids exhibit strong antibacterial property against gram positive bacteria and partial inhibition of gram-negative bacteria [26, 38]. In the present study, higher inhibition of gram negative *E*. *coli* was observed. An earlier study on Vetiver oil also reported high antibacterial activity against drug resistant strains of *E*. *coli* [37]. The antifungal activity of polyphenolic compounds is reported to be due to the formation of multinucleate stage by the breakage of *intersepta* in the mycelium and cell surface damage by pilferage [26].

Most of the antimicrobial drugs, particularly the antimycotic drugs, have several limitations such as low potency, poor solubility, emergence of resistant strains, and drug toxicity and have to be phased out with time [39]. As such, the search for new antimicrobial agents is necessary to stimulate the research of new therapeutic agents from medicinal plants. The present study also assumes importance as growth-promoting antibiotics in animal feeds are banned in European countries [40]. The detection of antioxidant property in *V*. *zizanioides* also indicates that it could be used in food supplements and nutraceuticals. There is increasing interest in using natural products including probiotics, prebiotics, enzymes, organic acids, and secondary plant metabolites or their nature-identical chemicals that do not pose a public health hazard as feed additives to solve problems in animal nutrition and livestock production and in future human diet.

## Figures and Tables

**Table 1:** Total phenolics and antioxidant values of different fractions of *V. zizanioides* leaves.

Phenolic fraction	Total phenolics∗ (GA equivalent *μ*M/g fwt)	Antioxidant property TEAC value (%)
HW	2.03 ± 0.9^b^	54.73
HM	0.97 ± 0.4^a^	21.45
NW	2.62 ± 1.7^c^	65.2
NM	1.09 ± 0.5^a^	26.63
M	1.19 ± 0.5^a^	30.04

^*^Values are mean ± standard error. Means followed by the same letter in a column are not significantly different at *P = 0.05* according to analysis of variance and Tukey's HSD test.

HW: water soluble acidic fraction, HM: methanol soluble acidic fraction, NW: water soluble alkaline fraction, NM: methanol soluble alkaline fraction, and M: 80% methanolic extract.

**Table 2:** Antifungal activity (antifungal index) of the phenolic extracts of *V. zizanioides* against the fungal strains *A*. *niger*, *F. oxysporum,* and *M*. *phaseolina*.

Phenolic fractions	Antifungal index∗ (%)		
	*A. niger *	*F. oxysporum *	*M. phaseolina *
HW	62.86 ± 1.65^ab^	64.79 ± 1.49^d^	67.11 ± 0.58^c^
HM	77.04 ± 0.46^d^	60.86 ± 0.66^c^	63.40 ± 0.88^bc^
NW	60.99 ± 0.98^a^	56.38 ± 1.31^b^	61.82 ± 1.95^b^
NM	81.20 ± 1.28^e^	59.89 ± 1.68^bc^	75.17 ± 0.91^d^
M	64.95 ± 0.81^b^	63.05 ± 1.12^cd^	71.80 ± 2.61^d^
Bavistin	71.26 ± 0.32^c^	38.77 ± 1.13^a^	52.24 ± 1.05^a^

^*^Values are mean ± standard error. Means followed by the same letter in a column are not significantly different at *P* = 0.05 according to analysis of variance and Tukey's HSD test.

HW: water soluble acidic fraction, HM: methanol soluble acidic fraction, NW: water soluble alkaline fraction, NM: methanol soluble alkaline fraction, and M: 80% methanolic extract.
